# ^18^F-AV45 PET-CT screening for systemic amyloidosis: A case report

**DOI:** 10.1097/MD.0000000000041871

**Published:** 2025-04-11

**Authors:** Bing Ruan, Haoxiang Zhu

**Affiliations:** a Zhuji People’s Hospital of Zhejiang Province, Shaoxing, China; b Department of Infectious Diseases, Huashan Hospital, Fudan University, Shanghai, China.

**Keywords:** ^18^F-AV45 PET-CT, abdominal wall fat biopsy, AL amyloidosis, myocardial amyloidosis

## Abstract

**Rationale:**

Systemic AL amyloidosis is a complex, multi-system disease with diverse clinical manifestations. Early and definite diagnosis helps to improve the prognosis.

**Patient concerns:**

At the beginning stage of the disease, the patient only presented with liver enlargement and abnormal liver function.

**Diagnoses:**

Conventional echocardiography “ECG” amyloidosis screening spot tracking imaging, etc. Pathology is the gold standard for diagnosis.

**Interventions:**

Elimination of clonal plasma cells or B cells that produce abnormal light chains. For eligible patients, autologous stem cell transplantation is the first choice.

**Outcomes:**

Systemic AL amyloidosis is often misdiagnosed due to a lack of typical symptoms and imaging signs.

**Lessons:**

This case highlights the patients with atypical clinical manifestations of AL amyloidosis, and the diagnosis should be made early to improve the prognosis. If there are risks and difficulties in pathological diagnosis, the cumulative organs can be indirectly evaluated with 18F-florbetapir positron emission tomography-computed tomography. Subsequent indirect diagnosis through less invasive, more accessible abdominal or rectal tissue.

## 1. Introduction

Systemic immunoglobulin light chain (AL) amyloidosis is a complex, multi-system disease with diverse clinical manifestations. Clinical diagnosis is difficult, but early diagnosis and treatment can improve the prognosis of this disease. Pathology is the gold standard for diagnosing amyloidosis, but biopsies of tissues such as the heart and liver carry significant risks. In a case of systemic amyloidosis primarily presenting with hepatomegaly and abnormal liver function, although the diagnosis of amyloidosis had been confirmed through liver biopsy, we still screened 18F-florbetapir positron emission tomography-computed tomography (^18^F-AV45 PET-CT) to assess organ involvement, and confirmed through abdominal fat biopsy that the amyloidosis was of the light-chain type. ^18^F-AV45 PET-CT, as a new diagnostic tool, can indicate amyloid deposits in tissues or organs, indirectly evaluate the extent of involvement, and assist in the early diagnosis of systemic amyloidosis.

## 2. Case report

A 77 years old retired male patient was admitted to the hospital due to “fatigue for more than a year, hepatosplenomegaly, and abnormal liver function for more than 6 months.” Initially, the patient exhibited only fatigue and weight loss. However, as the symptoms intensified, multiple liver function tests indicated mild elevations in alanine aminotransferase (ALT), aspartate aminotransferase (AST), and serum bilirubin, with alkaline phosphatase (ALP) showing significant elevation. Imaging studies revealed hepatosplenomegaly, including abdominal ultrasound, CT, and magnetic resonance imaging. Hepatic elastography indicated increased liver stiffness. An ^18^F-FDG PET-CT scan showed uniform radioactive distribution in the heart, liver, and spleen, with no abnormal foci of increased radioactive uptake. Mildly increased and uneven FDG metabolism was noted in the liver capsule, omentum, mesentery, and pelvic peritoneum, considering the possibility of inflammation. No apparent abnormalities were detected during the electronic colonoscopy. The patient was admitted to clarify the cause of the hepatosplenomegaly and liver function abnormalities. Medical history: The patient had a history of hepatitis A in 1975 and a 20-year history of alcohol consumption. He denied any family history of malignancies or genetic disorders. Upon admission, the physical examination revealed no jaundice in the skin or sclera, palmar erythema, spider angiomas, or palpable lymphadenopathy. The abdomen was slightly distended but soft, with no tenderness or rebound tenderness. The liver was palpated 3 to 4 fingerbreadths below the costal margin and was relatively firm; the spleen was not palpable. There was no shifting dullness, and bilateral lower extremities showed no edema.

After admission, routine blood tests mainly showed the expected results, but liver function tests indicated elevated levels of ALT (41 U/L), AST (80 U/L), total bilirubin (46.2 μmol/L), direct bilirubin (39.3 μmol/L), and ALP (363 U/L). Coagulation studies revealed a prolonged prothrombin time of 17.2 seconds and an international normalized ratio of 1.50.

**Figure 1.**
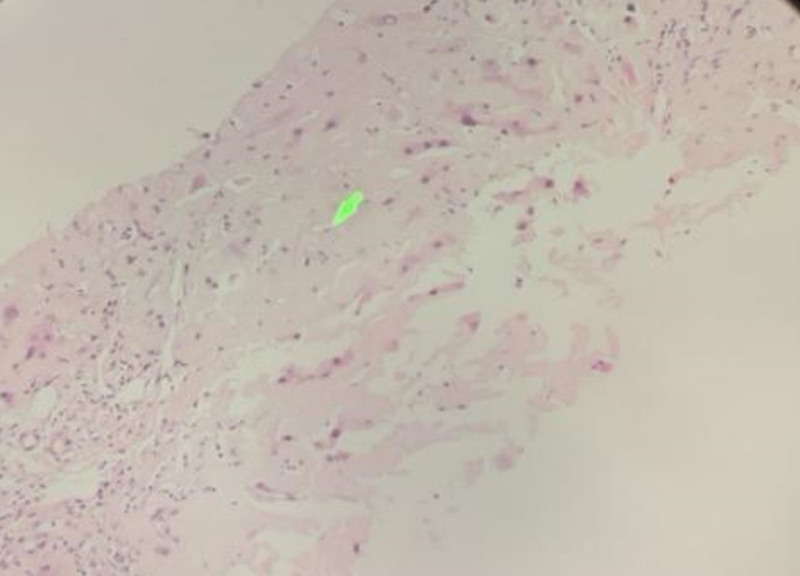
Pathology of liver puncture: a small amount of punctured tissue from the liver with large deposits of eosinophilic unstructured material in the hepatic sinusoids, consistent with amyloidosis.

**Figure 2.**
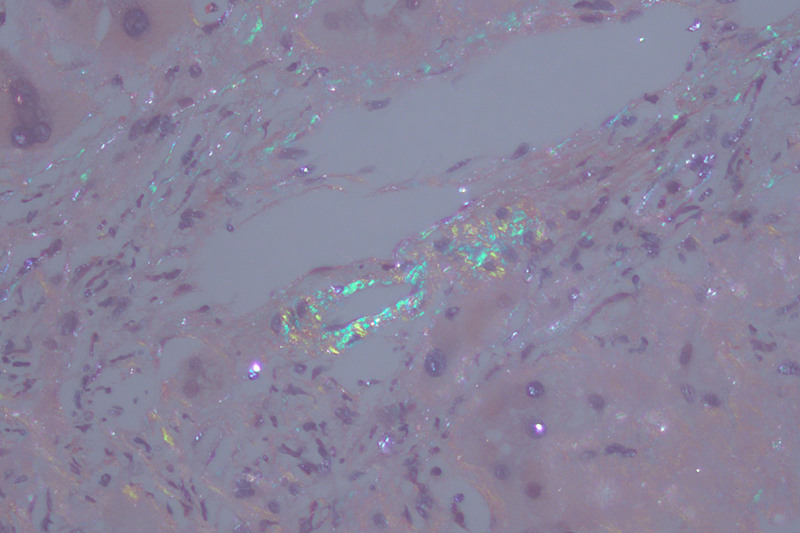
Polarized light microscopy of liver tissue showing typical birefringence.

Because of hepatomegaly and coagulation abnormalities, percutaneous liver puncture biopsy was risky, and transjugular liver puncture biopsy was perfected after communication with the family. Liver pathology suggested that a large amount of eosin unstructured material was deposited in the hepatic sinusoids, positive Congo red staining, and typical birefringence visible under polarized light microscopy, consistent with amyloidosis (Figs. 1 and 2). Since amyloidosis is most common in light chain type, we conducted blood and urine immunofixation electrophoresis and free light chains, resulted with Bence-Jones protein Kappa light chain positive in urine immunofixation electrophoresis, serum free κ/λ: 14.82 (0.31–1.56).1% abnormal plasma cells were seen in bone marrow smear.

**Figure 3.**
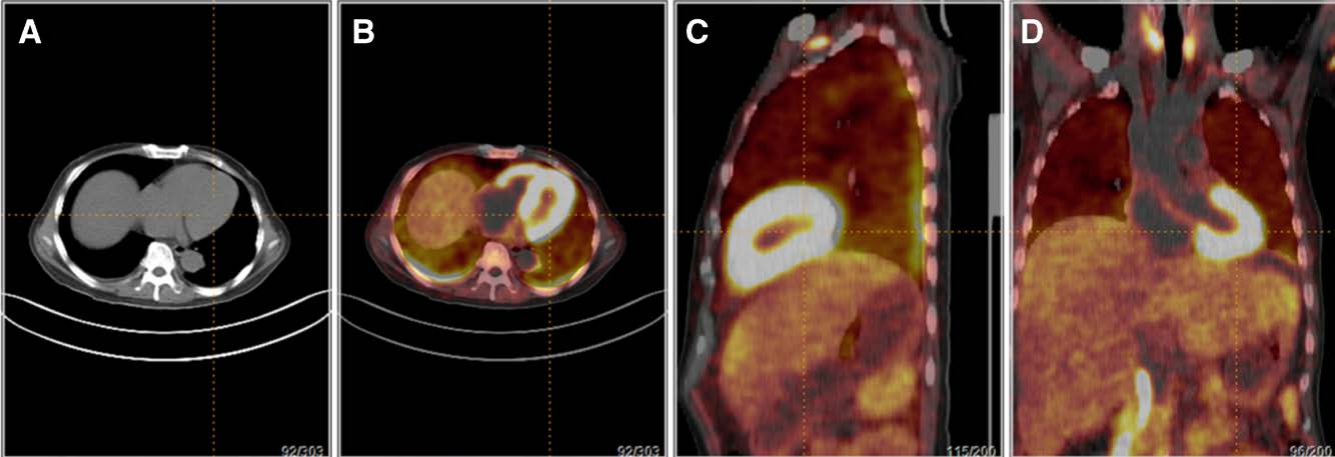
18F-AV45 PET-CT: unevenly increased radiotracer uptake is observed in the heart, liver, spleen, omentum, mesentery, pelvic peritoneum, and bone marrow.

To further evaluate the involved organs, ^18^F-AV45 PET-CT was performed, which suggested diffuse increase in radioactivity uptake in the right and left ventricles, with a maximum standardized uptake value (SUV) value of 11.8; diffuse enlargement of the liver with unevenly increased radioactivity uptake, with a maximum SUV value of 4.6; uneven thickening of the greater omentum, mesentery, and pelvic peritoneum with unevenly increased radioactivity uptake, with a maximum SUV value of 4.2; diffuse increase in radioactivity uptake in the spleen, with a maximum SUV value of 4.1; unevenly increased radioactivity uptake in bone marrow, with a maximum SUV value of 3.8. Diffusely increased radioactivity uptake in the spleen with a maximum SUV of 4.1; unevenly increased radioactivity uptake in the bone marrow with a maximum SUV of 3.8. Combined with the history, amyloidosis was considered to be multisystemic in nature (Fig. 3). AV45 PET-CT suggested cardiac involvement, complete cardiac evaluation, no electrocardiogram abnormalities; troponin T 0.075 ng/mL, creatine kinase ioszyme mass 8.21 ng/mL, pro-brain natriuretic peptide (BNP) 5066 pg/mL; echocardiography: severe hypo-diastolic function of the left heart; cardiac amyloidosis screening speckle-tracking imaging analysis: overall longitudinal strain in the left ventricle was significantly reduced, bull’s-eye image showed preserved longitudinal strain in the left ventricular apical region, consistent with the manifestation of cardiac amyloidosis.

Since amyloidosis often involves the kidneys, the urine routine was performed: urinary bilirubin 1+, bilirubin 1+, urinary protein 1+; 24-hour urinary protein quantification: 0.28 g; renal function: urea nitrogen 7.2 mmol/L, creatinine 60 umol/L; renal ultrasound did not show apparent abnormalities, and the evidence for the amyloidosis involving kidney was insufficient. In order to clarify the amyloidosis typing, the pathology of the abdominal fat biopsy further showed subcutaneous adipose tissue amyloidosis (inclined to AL type), Congo red staining positive, kappa brightness slightly stronger than lambda, and evident birefringence phenomenon was seen under polarized light microscope.

Based on these findings, the patient was diagnosed systemic light chain amyloidosis (AL type), which affected the heart, liver, and adipose tissue. The patient was transferred to the hematology department for ongoing treatment and received 4 cycles of chemotherapy with dexamethasone and daratumumab. After the chemotherapy, the patient’s ALT, AST, and total bilirubin levels stabilized. A slight decrease in ALP and a gradual decline in serum-free light chains and pro-BNP indicated that the condition was essentially stable. However, 1 month later, the patient experienced a rapid increase in inflammatory markers due to an infection. This led to unstable blood pressure, decreased oxygen saturation, markedly elevated pro-BNP, and a sharp rise in creatinine levels. Septic shock and rapid deterioration of cardiac and renal functions were suspected. Despite aggressive interventions, including intubation for mechanical ventilation, blood transfusions, antibiotic treatment, and continuous renal replacement therapy, the patient’s condition did not improve, resulting in his death on November 1st.

## 3. Discussion

Amyloidosis is a collective term for a group of clinical disorders in which amyloidogenic proteins misfold to form antiparallel β-folded sheet structures that are deposited in human tissues and organs, causing organ dysfunction.^[1]^ Systemic amyloidosis of the light chain (AL) type is the most common in the clinic, which is a disease in which monoclonal immunoglobulin light chain misfolds to form amyloid and deposits in tissues and organs, resulting in tissue structural damage, organ dysfunction, and progressive progression, mainly related to clonal plasma cell proliferation, and to a lesser extent, related to lymphoproliferative disorders. It has been reported^[2]^ that the heart is affected in more than 75% of patients, the kidney in more than 60%, and other organs, including the liver, autonomic or peripheral nerves, gastrointestinal tract, and soft tissues of the skin. Clinical manifestations include heart failure, nephrotic syndrome, proteinuria, hepatomegaly, peripheral neuropathy, periorbital purpura, and tongue enlargement. The clinical manifestations of amyloidosis involving the liver are usually atypical and easily misdiagnosed. The case reported in this article mainly showed atypical symptoms such as malaise, hepatosplenomegaly, mild liver function abnormality, and no symptoms of other systemic involvement such as chest tightness, shortness of breath, telangiectasias, lower limb edema, foamy urine, impaired renal function, etc. The diagnosis of this patient was unclear after several routine investigations, and there was an indication for liver biopsy. However, the patient’s liver was obviously enlarged, and percutaneous liver puncture biopsy had a high risk of bleeding. After communication with the family, transjugular hepatic puncture was perfected, and the final pathology showed typical changes of hepatic amyloidosis, which confirmed this diagnosis. According to the literature studied by Miguel A. Park,^[3]^ 98 cases were confirmed as hepatic amyloidosis by liver biopsy. However, patients with amyloidosis usually have obvious liver enlargement, and liver puncture carries the risk of liver rupture or hemorrhage and has been reported to be fatal and the technique of transjugular vein liver biopsy has not been comprehensively performed at present, which makes the diagnosis of this disease difficult.

^18^F-AV 45 reversibly binds to the portion of the amyloid fibril component that undergoes β-folding, showing high affinity and the site of amyloid deposition exhibits increased uptake values. In this case, although the amyloidosis was clearly defined by liver histopathology, ^18^F-AV 45 PET-CT was still performed to evaluate the involvement of other organs, and the results revealed heterogeneous thickening of the right and left ventricles, liver and spleen, bone marrow, greater omentum, mesentery, and pelvic floor peritoneum, accompanied by increased AV45 uptake. AV45 PET-CT suggests obvious cardiac involvement, regargless of no chest tightness, shortness of breath and other signs of cardiac insufficiency, we still took the traditional noninvasive examination to assist in the evaluation of the patient, the patient’s N-terminal pro-brain natriuretic propeptide and troponin abnormalities, and echocardiography and “bull’s-eye diagram” have characteristic manifestations, which is consistent with the diagnosis of cardiac amyloidosis. The patient’s NTpro-BNP and troponin abnormalities and characteristic echocardiographic and “bull’s-eye” findings were consistent with a diagnosis of cardiac amyloidosis. ^18^F-AV 45 PET-CT has been used in the past decades for the auxiliary diagnosis of Alzheimer disease,^[4]^ but in our hospital, ^18^F-AV 45 PET-CT has been carried out for the diagnosis and assessment of amyloidosis. ^18^F-AV 45 PET-CT can help evaluate the involved sites in patients with amyloidosis, and it was found that majority of patients with amyloidosis had significantly elevated SUV values at the site of involvement. In our patient, there were no significantly elevated SUV sites in the conventional ^18^FDG-metabolized PET-CT, and elevated SUV values could be seen in multiple sites, including the heart, bone marrow, liver, spleen, and omentum when switched to ^18^F-AV 45 PET-CT, suggesting it is useful in screening for amyloidosis.

Tissue biopsy is always the gold standard for the diagnosis of amyloidosis. If the risk of liver or heart puncture is too high, we can choose the safer rectal mucosal or abdominal fat biopsy. It is relatively less invasive, has fewer complications, and can be used to diagnose amyloidosis, also for direct typing of amyloid proteins, with a sensitivity of up to 79% for AL-type amyloidosis and even up to 100% for biopsies when the sampling area is more significant than 700 mm^3^.^[5]^ The combination with bone marrow biopsy can improve the positive diagnosis rate. In this case, although we had diagnosed amyloidosis by liver puncture, we just could not differentiate the type of amyloidosis. Then, we differentiated the type of amyloidosis by abdominal wall fat biopsy and finally diagnosed AL-type amyloidosis.

Treatment of AL amyloidosis focuses on removing plasma cells or B-cell clones that produce abnormal light chains. Transplantation is preferred if the patient is eligible for autologous hematopoietic stem cell transplantation, and anti-plasma cell therapy is used if the patient is not eligible for transplantation. The most commonly regimen is the combination of cyclophosphamide/bortezomib/dexamethasone. A regimen of daratumumab (DARA) in combination with cyclophosphamide/bortezomib/dexamethasone is also available. If this regimen is not tolerated, DARA alone or combined with dexamethasone may be used.^[6]^ Autologous hematopoietic stem cell transplantation may also be followed by symptom improvement after anti-plasmacytosis therapy.^[7]^ The prognosis of this disease is poor, and the mostly causes of death are heart or renal failure. The chemotherapy regimen of DARA combined with dexamethasone was chosen for the initial treatment in our case. In the initial phase of chemotherapy, the patient’s serum free light chain decreased significantly, but liver function did not improve significantly, and hepatomegaly did not resolve. Eventually, severe septic shock developed due to infection. Although cardiac and renal involvement was not apparent at the early stage of amyloidosis, acute renal failure and cardiac failure developed rapidly after infection, which caused the rapid death of this patient.

## 4. Conclusions

In conclusion, systemic light chain amyloidosis is a complex multidisciplinary disease, often involving multiple systems, with diverse and atypical clinical manifestations, making it easy to misdiagnose, and pathology is the gold standard for diagnosis. However, biopsy of tissues such as heart and liver carries a higher risk, and abdominal fat biopsy may be a good choice. It is worth mentioning that ^18^F-AV45 PET-CT can be used as a noninvasive screening tool for patients with clinically suspected amyloidosis.

## Author contributions

**Data curation:** Bing Ruan.

**Formal analysis:** Bing Ruan.

**Methodology:** Haoxiang Zhu.

**Resources:** Bing Ruan.

**Validation:** Haoxiang Zhu.

**Visualization:** Haoxiang Zhu.

**Writing – original draft:** Bing Ruan.

**Writing – review & editing:** Haoxiang Zhu.
